# Infants fed formula with added long chain polyunsaturated fatty acids have reduced incidence of respiratory illnesses and diarrhea during the first year of life

**DOI:** 10.1186/1471-2431-14-168

**Published:** 2014-07-02

**Authors:** Alexandre Lapillonne, Nitida Pastor, Weihong Zhuang, Deolinda MF Scalabrin

**Affiliations:** 1 Department of Neonatology, APHP Necker Enfants Malades Hospital, Paris Descartes University, Paris, France; 2 Department of Medical Affairs, Clinical Research, Mead Johnson Nutrition, Evansville, USA

**Keywords:** DHA, ARA, LCPUFAs, Infant, Infant formula, Infant nutrition, Respiratory illness, Diarrhea

## Abstract

**Background:**

Long chain polyunsaturated fatty acids (LCPUFAs) may influence the immune system. Our objective was to compare the frequency of common illnesses in infants who received formula with or without added LCPUFAs.

**Methods:**

In this observational, multi-center, prospective study, infants consumed formula with 17 mg DHA and 34 mg ARA/100 kcal (n = 233) or with no added DHA or ARA (n = 92). Pediatricians recorded respiratory illnesses, otitis media, eczema, and diarrhea through 1 year of age.

**Results:**

Infants who consumed formula with DHA/ARA had lower incidence of bronchitis/bronchiolitis (*P* = 0.004), croup (*P* = 0.044), nasal congestion (*P* = 0.001), cough (*P* = 0.014), and diarrhea requiring medical attention (*P* = 0.034). The odds ratio (OR) of having at least one episode of bronchitis/bronchiolitis (0.41, 95% CI 0.24, 0.70; *P* = 0.001), croup (0.23, 95% CI 0.05, 0.97; *P* = 0.045), nasal congestion (0.37, 95% CI 0.20, 0.66; *P* = 0.001), cough (0.52, 95% CI 0.32, 0.86; *P* = 0.011), and diarrhea requiring medical attention (0.51, 95% CI 0.28, 0.92; *P* = 0.026) was lower in infants fed DHA/ARA. The OR of an increased number of episodes of bronchitis/bronchiolitis, croup, nasal congestion, cough, and diarrhea, as well as the hazard ratio for shorter time to first episode of bronchitis/bronchiolitis, nasal congestion, cough, and diarrhea were also significantly lower in the DHA/ARA group.

**Conclusions:**

In healthy infants, formula with DHA/ARA was associated with lower incidence of common respiratory symptoms and illnesses, as well as diarrhea.

## Background

Appropriate nutrition during infancy and early childhood provides critical support to the immune system and may reduce the incidence of common illnesses during this age period. Attention has increasingly focused on the potential role of long-chain polyunsaturated fatty acids (LCPUFAs) such as docosahexaenoic acid (DHA; 22:6n-3) and arachidonic acid (ARA; 20:4n-6) as immunomodulatory nutrients [1]. Infants receive LCPUFAs from dietary sources including human milk, infant formula with added DHA and ARA, certain types of food rich in LCPUFAs such as fish, fish oil, and eggs, and endogenously through the conversion of the precursors alpha-linolenic and linoleic acid. DHA and ARA are incorporated into cell membranes and contribute to immune cell activity through a variety of different mechanisms [2]. In previous studies, both preterm [3] and full-term [4] infants who were fed formula with DHA and ARA displayed lymphocyte populations, cytokine production, and immune cell maturation that resembled those of breastfed infants, suggesting that dietary intake of DHA and ARA, whether via human milk or infant formula, contributes to immune development.

Dietary LCPUFAs have been shown to affect the incidence of respiratory illnesses and allergic manifestations during infancy and childhood [5]. Higher concentrations of LCPUFAs in human milk, for example, were associated with reduced incidence of atopic diseases [6,7], while maternal supplementation with LCPUFAs during lactation was associated with a reduction in the incidence of bronchopulmonary dysplasia and allergic rhinitis in preterm infants [8]. Additionally, early introduction of fish has been associated with reduced prevalence of eczema [9-11], allergic rhinitis [12,13], and recurrent wheezing [14]. Few studies, however, have specifically investigated the effects of infant formula with DHA and ARA on such illnesses. In an observational study in Spain, infant formula with DHA and ARA was associated with a lower incidence of respiratory illnesses [15]. Based on this observation, we designed the current study to compare the frequency of common illnesses during the first year of life in healthy infants receiving formula with or without added DHA and ARA. We sought to confirm our prior results on respiratory illnesses [15], in a different population of infants, and to extend those results by tracking the incidence of other common infant illnesses such as otitis media, diarrhea, and eczema.

## Methods

### Study design

Enrollment for this multicenter, prospective, observational, open-label study was conducted from January 2008 to April 2009 at 22 pediatric outpatient clinics in France. Inclusion criteria were: healthy term infants born after 37 weeks of gestation, singleton, with a birth weight, length, and head circumference appropriate for gestational age (≥10th and ≤90th percentile), less than 60 days of age at enrollment, and exclusively fed with one of the study formulas for at least 24 hours before enrollment. Exclusion criterion was participation in any other clinical trial.

Infants were categorized into one of two study groups based on the formula they were currently consuming:

•DHA/ARA: infant and follow-on formulas containing 17 mg of DHA/100 kcal and 34 mg of ARA/100 kcal (Enfamil Premium 1^®^ and Enfamil Premium 2^®^, Mead Johnson Nutrition, Evansville, IN, USA) or

•Control: infant and follow-on formulas without added DHA/ARA (Enfalac^®^ and Enfamil 2^®^, Mead Johnson Nutrition, Evansville, IN, USA).

The composition of the formulas in the 2 groups was similar; the main difference between them was the content of DHA and ARA. The infants received infant formulas until approximately 4 months of age (i.e. Enfamil Premium 1^®^ or Enfalac^®^) and follow-on formulas (Enfamil Premium 2^®^ or Enfamil 2^®^) from approximately 4 months until 12 months of age. Recommendations with regards to the introduction of weaning foods were left to the discretion of the pediatrician.

Each pediatrician participating in this study was estimated to have the capacity to enroll 4–6 eligible infants per month. Participating pediatricians were asked to enroll eligible infants that were already consuming one of the pre-determined infant formulas. The choice of formula was made at the discretion of the parents before the recruitment. The pediatrician was instructed to not recommend a formula other than the one already being used by the participant at the time of enrollment.

Pediatricians followed participants until 12 months of age at routine visits and any unscheduled visit or telephone call. Routine visits were at 1 to 2 months (visit 1), 2 to 3 months (visit 2), and approximately 4 (visit 3), 6 (visit 4), and 12 (visit 5) months of age.

At the initial visit, the pediatrician completed a case report form including information on the infant’s medical history, family history of allergy, and parents’ socioeconomic background. The case report form provided 7 categories in which to record parents’ professional information. Among them, “No professional activity” was the lower level; “Farmers”, “Craftsmen, businessmen, business owners”, “Intermediate professions”, “Office employees”, and “Labors” were combined to form an intermediate level; “Upper level management and higher-level professions” composed the upper level. Six levels of socioeconomic status were then formed using a combination of both parents’ socioeconomic status, e.g.: both parents upper level; one upper level and one intermediate level; etc.

At each visit, anthropometric measurements and occurrence of feeding intolerance were recorded, as well as compliance with the study formulas. Respiratory symptoms and illnesses (nasal congestion, cough, bronchitis/bronchiolitis, and croup), otitis media, diarrhea, and eczema, as assessed by the pediatrician, were also recorded. At each visit, anthropometric variables were recorded and converted to z-scores based on WHO references [16].

### Ethics

This study was conducted according to the guidelines of the Declaration of Helsinki and all procedures involving human subjects/patients and handling of medical records were approved by the appropriate local authorities [National Board of Physicians (CNOM), the Consultative Committee on Information Processing in Health Research (CCTIRS) and the National Commission on Informatics and Liberties (CNIL)]. All parents were given written information on the study and their informed verbal consent was obtained in all cases. Each patient was monitored with no obligation or constraint; in particular, no follow-up visit, medical procedure, or additional exam of any sort was imposed. The participating pediatricians remained free to choose the medical treatment for each participant’s events. This research has adhered to the guidelines for qualitative research review (RATS) [17].

### Statistical analysis

Characteristics of the study population, including family history of eczema, asthma, and bronchiolitis, smoking in the home, gender and type of feeding (breastfed or non-breastfed) prior to study formula were analyzed using Fisher's exact test. Differences in duration of breastfeeding for those who were breastfed were analyzed by analysis of variance (ANOVA). Weight, length, and head circumference at birth were analyzed for males and females separately by ANOVA. For each type of illness, the proportion of infants in each group having at least one episode during the first year of life was compared using Fisher’s exact test.

To adjust for covariates, multiple logistic regression was used to examine the association between formula type and illness. For infection-related illnesses or symptoms of illness (bronchitis/bronchiolitis, nasal congestion, cough, croup, otitis media, and diarrhea requiring medical attention), potential covariates included mother’s educational level, father’s educational level, parents’ socioeconomic status, smoking in the home, number of people living in the home, and daycare exposure. Potential covariates for allergy-related illness (eczema) included gender, family allergy history, mother’s educational level, father’s educational level, parents’ socioeconomic status, smoking in the home, number of people living in the home, and daycare exposure. All potential covariates were retained in a preliminary logistic regression model and stepwise selection was used to select covariates with significant evidence at an alpha level of 0.05. The selected covariates were then included in a final logistic regression model.

Further analyses using an ordinal model examined the number of episodes of illness or symptom. For the ordinal analysis of bronchitis/bronchiolitis, cough, and nasal congestion, the number of episodes was truncated at 3, due to sparse frequency of more than 3 episodes. For the ordinal analysis of all other illnesses, the number of episodes was truncated at 2. The same set of covariates was included in the ordinal model as were used in the final logistic regression model.

In the multiple regression analysis and the ordinal analysis, only two covariates reached significance (mother’s educational level and early daycare exposure) and for only one outcome (bronchiolitis/bronchitis). A Cox proportional hazards model was used to examine the time to first diagnosis of illness during the study period.

All *P* values reported are based on two-tailed tests. A *P* value of < 0.05 was considered statistically significant for all analyses. Statistical analyses were performed using SAS^®^ software (version 9.1; SAS Institute, Cary, NC).

## Results

### Participant characteristics

Three hundred and twenty five infants were included in the study. Infant characteristics at birth were similar between groups (Table 1) with the exception of weight (Mean ± SE; Control: 3195 ± 68.3 g, DHA/ARA: 3383 ± 41.4 g; *P* = 0.02) and length (Control: 48.7 ± 0.3 cm, DHA/ARA: 49.6 ± 0.2 cm; *P* = 0.04) of females, which were significantly lower in the Control group, but these differences were no longer significant at the time of study enrollment. The number of infants who were reported as being breastfed at any time prior to study enrollment was significantly higher in the control group (n (%); Control: 70 (76%); DHA/ARA 144 (62%); *P* = 0.02); however, when feeding type was further broken down into the 3 categories of non-breastfed, mixed feeding, and exclusively breastfed, there was no significant difference in the distribution between Control and DHA/ARA groups (*P* = 0.07; Table 1). The duration (days) of breastfeeding for those infants who were breastfed was not different in the 2 groups (Mean ± SE; Control: 34 ± 2.3; DHA/ARA: 32 ± 1.5; *P* = 0.52). The number of infants with early daycare exposure was higher in the DHA/ARA group, but there was no difference in late daycare exposure (Table 1).

**Table 1:** Characteristics of the study participants

	**DHA/ARA**	**Control**	***P*-value**
Study enrollment, n	233	92	—
Study completion, n (%)	204 (88%)	78 (85%)	0.59
Gender (male), n (%)	121 (52%)	51 (55%)	0.62
**Birth characteristics**			
Weight (g), Mean (SE)			
*Males (DHA/ARA n = 119; Control n = 51)*	3375 (39.1)	3433 (59.7)	0.42
*Females (DHA/ARA n = 109; Control n = 40)*	3383 (41.4)	3195 (68.3)	0.02
Length (cm), Mean (SE)			
*Males*	49.9 (0.2)	50.2 (0.3)	0.41
*Females*	49.6 (0.2)	48.7 (0.3)	0.04
Head circumference (cm), Mean (SE)			
*Males*	35.0 (0.1)	34.9 (0.2)	0.65
*Females*	34.6 (0.1)	34.3 (0.2)	0.13
**Enrollment characteristics**			
Age (days) at enrollment			
*Males*	41.3 (1.5)	38.3 (2.3)	0.28
*Females*	45.6 (1.4)	43.2 (2.4)	0.39
Weight (g), Mean (SE)			
*Males*	4594.9 (75.2)	4524.1 (113.9)	0.61
*Females*	4566.8 (68.8)	4378.4 (117.0)	0.17
Length (cm), Mean (SE)			
*Males*	55.0 (0.3)	54.7 (0.4)	0.53
*Females*	54.9 (0.3)	54.3 (0.4)	0.28
Head circumference (cm), Mean (SE)			
*Males*	38.0 (0.2)	37.7 (0.2)	0.32
*Females*	37.7 (0.1)	37.4 (0.2)	0.26
Feeding			
*Ever breastfed, n (%)*	144 (62%)	70 (76%)	0.02
*Non-breastfed, n (%)*	88 (43%)	22 (30%)	
*Mixed feeding, n (%)*	60 (29%)	21 (28%)	0.07
*Exclusively breastfed, n (%)*	59 (29%)	31 (42%)	
*Breastfeeding duration (days), Mean (SE)*	32 (1.5)	34 (2.3)	0.52
Family history of allergy, n (%)			
*Eczema*	49 (21%)	19 (21%)	1.00
*Asthma*	47 (20%)	16 (17%)	0.64
Smoking in home, n (%)	50 (22%)	21 (23%)	0.88
Mother’s educational level, n (%)			0.54
*High*	64 (28%)	20 (22%)	
*Intermediate*	142 (63%)	60 (67%)	
*Low*	19 (8%)	9 (10%)	
Parents’ socioeconomic level, n (%)			0.80
*Both upper*	36 (16%)	15 (16%)	
*One upper/One intermediate*	32 (14%)	13 (14%)	
*One upper/One lower*	9 (4%)	1 (1%)	
*Both intermediate*	111 (49%)	46 (51%)	
*One intermediate/One lower*	35 (15%)	16 (18%)	
*Both lower*	3 (1%)	0 (0%)	
Daycare attendance, n (%)			
*Early daycare exposure (≤90 days of age)*	12 (5%)	0 (0%)	0.02
*Late daycare exposure*	99 (45%)	36 (42%)	0.61

Gender, family history of eczema, asthma, and bronchiolitis, and smoking in the home, mother’s educational level, and type of feeding were analyzed using Fisher's exact test. All other variables were analyzed by ANOVA; statistical significance at *P* < 0.05.

### Growth

Group z-scores for females differed for weight-for-age at visit 4 (approx. 6 months of age) (Mean ± SE; Control: −0.2 ± 0.1; DHA/ARA: 0.2 ± 0.1; *P* = 0.015) and visit 5 (approx. 9 months of age) (Mean ± SE; Control: 0.0 ± 0.1; DHA/ARA: 0.5 ± 0.1; *P* = 0.005). There were no significant differences in length- or head circumference-for-age z-scores for females and in weight-, length-, and head circumference-for-age z-scores for males at any time during the study (data not shown).

### Incidence of illnesses

**Figure 1:**
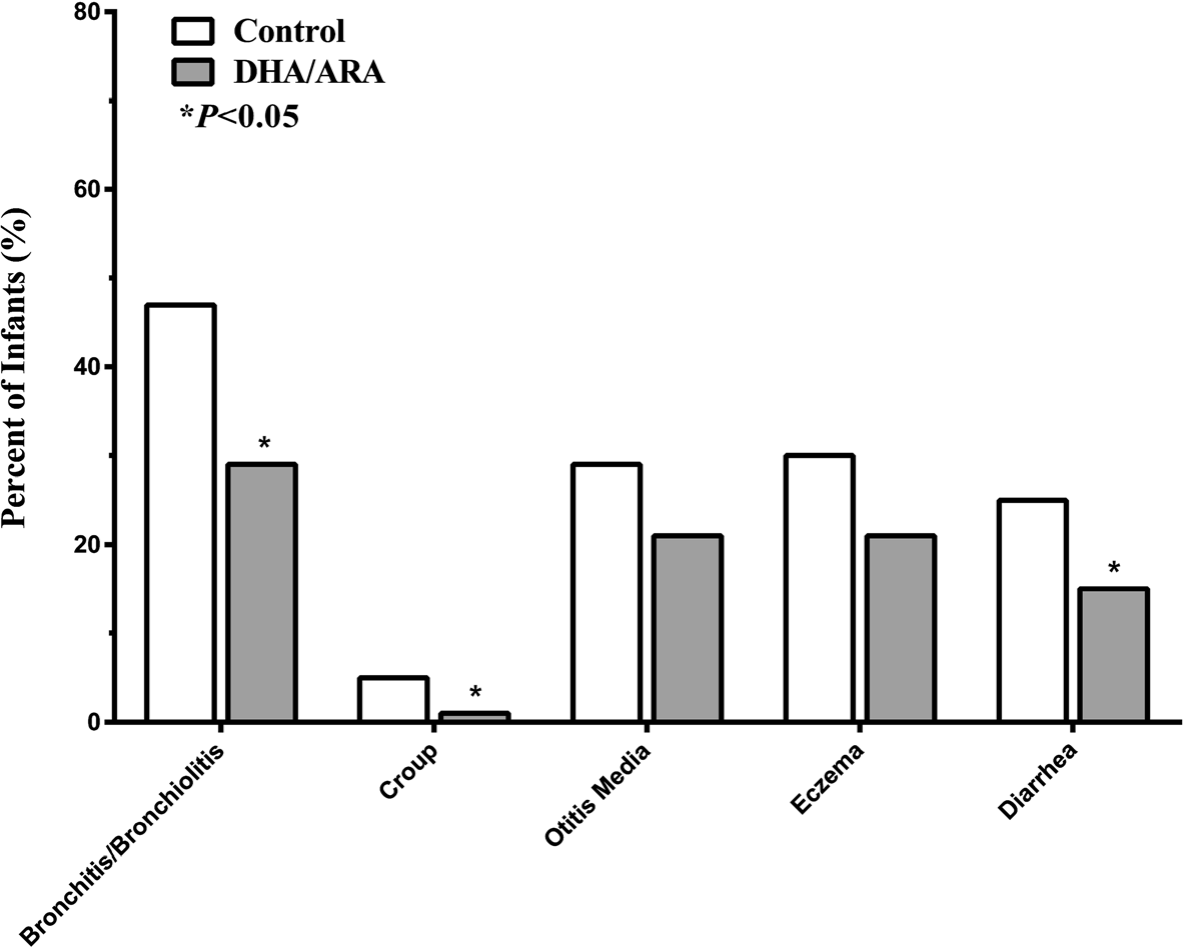
**Incidence of common illnesses during the first year of life.** Incidence of at least one episode of illness in healthy term infants during the first year of life, according to type of formula (with or without DHA/ARA). For each type of illness, the proportion of infants having at least one episode during the first year of life was compared between groups using Fisher’s exact test.

DHA/ARA consumption was associated with a lower incidence (n, %) of respiratory illnesses, with a significant effect on both bronchitis/bronchiolitis (Control: 43 (47%), DHA/ARA: 68 (29%); *P* = 0.004) and croup (Control: 5 (5%), DHA/ARA: 3 (1%); *P* = 0.044) (Figure 1). DHA/ARA intake was also associated with a lower incidence (n, %) of symptoms of respiratory illness during the first year of life, including nasal congestion (Control: 75 (82%), DHA/ARA: 144 (62%); *P* = 0.001) and cough (Control: 58 (63%), DHA/ARA: 110 (47%); *P* = 0.014).

With regard to other common infant illnesses, DHA/ARA intake was associated with a lower incidence (n, %) of diarrhea requiring medical attention (Control: 23, (25%), DHA/ARA: 34 (15%); *P* = 0.034), but there was no statistically significant difference in the incidence of eczema (Control: 28 (30%), DHA/ARA: 49 (21%); P = 0.083) or otitis media (Control: 27 (29%), DHA/ARA: 48 (21%); *P* = 0.109) (Figure 1).

The odds ratio (OR) of having at least one episode of bronchitis/bronchiolitis, croup, nasal congestion, cough, and diarrhea requiring medical attention was significantly lower for the DHA/ARA versus Control group (Table 2). The OR of increased number of episodes of bronchitis/bronchiolitis, croup, nasal congestion, cough, and eczema, as well as diarrhea requiring medical attention was also significantly lower in the DHA/ARA formula group (Table 3). In addition, the hazard ratio (HR) of time to first episode of bronchitis/bronchiolitis, nasal congestion, cough, and diarrhea requiring medical attention was significantly lower in the infants fed formula with DHA/ARA (Table 4).

**Table 2:** OR of having at least one episode of common illnesses in the DHA/ARA group compared to control

		**OR**	**95% CI**	***P*-value**
**Respiratory illness**	Bronchitis/Bronchiolitis^a^	0.41	(0.24, 0.70)	0.001
	Croup	0.23	(0.05, 0.97)	0.045
**Symptoms of respiratory illness**	Nasal Congestion	0.37	(0.20, 0.66)	0.001
	Cough	0.52	(0.32, 0.86)	0.011
**Other illnesses**	Otitis Media	0.63	(0.36, 1.09)	0.097
	Eczema	0.60	(0.34, 1.04)	0.067
	Diarrhea	0.51	(0.28, 0.92)	0.026

OR obtained from multiple logistic regression analysis; ^a^Bronchitis/Bronchiolitis was adjusted for the covariates early daycare exposure and mother’s educational level; no other covariates reached significance for any other illnesses; statistical significance at *P* < 0.05.

**Table 3:** OR of having increased number of episodes of common illnesses in the DHA/ARA group compared to control

		**OR**	**95% CI**	***P*-value**
**Respiratory illness**	Bronchitis/Bronchiolitis^a^	0.36	(0.22, 0.59)	0.001
	Croup	0.23	(0.05, 0.97)	0.045
**Symptoms of respiratory illness**	Nasal Congestion	0.45	(0.29, 0.69)	0.001
	Cough	0.47	(0.30, 0.74)	0.001
**Other illnesses**	Otitis Media	0.62	(0.36, 1.07)	0.084
	Eczema	0.57	(0.33, 0.98)	0.043
	Diarrhea	0.50	(0.27, 0.90)	0.021

OR obtained from ordinal model analysis; ^a^Bronchitis/Bronchiolitis was adjusted for the covariates early daycare exposure and mother’s educational level; no other covariates reached significance for any other illnesses; statistical significance at *P* < 0.05.

**Table 4:** HR for shorter time to first episode of common illnesses in the DHA/ARA group compared to control

		**HR**	**95% CI**	***P*-value**
**Respiratory illness**	Bronchitis/Bronchiolitis	0.52	(0.36, 0.77)	0.001
	Croup	0.24	(0.06, 1.00)	0.050
**Symptoms of respiratory illness**	Nasal Congestion	0.72	(0.54, 0.95)	0.023
	Cough	0.62	(0.45, 0.85)	0.003
**Other illnesses**	Otitis Media	0.67	(0.42, 1.07)	0.096
	Eczema	0.70	(0.44, 1.11)	0.127
	Diarrhea	0.55	(0.32, 0.93)	0.026

HR obtained from Cox proportional analysis; no covariates reached significance for any illnesses; statistical significance at *P* < 0.05.

## Discussion

In this study, we observed that infants fed infant formula with DHA and ARA had a lower incidence and delayed onset of respiratory illnesses and symptoms of respiratory illnesses, as well as diarrhea requiring medical attention, when compared to infants who received formula without DHA and ARA.

The previous observational study that we conducted in Spain indicated that consumption of infant formula with DHA and ARA was associated with a reduction in respiratory illnesses in infants during the first year of life, with significantly lower incidence of bronchitis/bronchiolitis at 5, 7, and 9 months of age, and upper respiratory infections at 1 and 12 months of age [15]. In the current study, we observed a similar, significant reduction in the cumulative incidence of bronchitis/bronchiolitis and croup during the first year of life. Infants fed formula with DHA and ARA also had a lower risk, fewer recurrent episodes, and delayed onset of bronchitis/bronchiolitis. They were also less likely to have one or more episodes of croup or multiple episodes of eczema. These results support our previous observation in a different infant population and are consistent with data showing that DHA and ARA impact immune function and inflammatory responses.

The results are consistent with other reports of a lower incidence of respiratory illnesses and symptoms among infants who consumed infant formula with DHA and ARA, including a retrospective review of medical records of infants that participated in two double-blind, randomized, clinical trials in the United States [18]. The retrospective review indicated that infants fed formula with DHA and ARA throughout the first year of life had a lower incidence and delayed onset of upper respiratory infections and common allergic manifestations during the first 3 years of life, compared with infants fed formula without DHA and ARA. Similar to our current observation, the retrospective review showed no effect of DHA/ARA on otitis media. Overall, these findings are consistent with the hypothesis that daily intake of LCPUFAs during the first year of life supports the developing immune system.

Our results are also aligned with studies that have examined the potential benefits of dietary LCPUFAs in older children. For example, in a double-blind, randomized clinical trial in children 18–36 months of age in the United States primarily designed to detect changes in DHA status, consumption of single daily serving of a cow’s milk-based beverage containing DHA (130 mg) for 2 months was associated with a significantly lower incidence of respiratory illnesses compared to the control beverage with no DHA [19]. Additionally, a study in Thai school children found that 9 to 12 year-old children who received milk with fish oil for 6 months had lower incidence, fewer episodes, and shorter duration of illnesses, including respiratory infections [20].

LCPUFAs may influence immune cell function through a number of mechanisms related to membrane composition, cell signalling, and gene regulation, among others [2]. Additionally, LCPUFA-derived lipid mediators appear to have anti-inflammatory properties and hasten the resolution of inflammation [21-23], which is consistent with clinical studies demonstrating that inflammatory conditions may be reduced or prevented by LCPUFAs [15,18-20,24-26].

We also found that the infants fed the DHA/ARA formula had a lower risk, decreased number of episodes, delayed onset, and a lower incidence of diarrhea requiring medical attention during the first year of life. Interestingly, a recent study in India demonstrated a shorter duration of episodes of mild gastrointestinal symptoms such as lack of appetite or abdominal pain in children (ages 6–10) who consumed a food supplement fortified with omega-3 fatty acids for a period of 12 months [27]. It has been previously suggested that omega-3 fatty acids such as DHA may reduce gastrointestinal inflammation [28], and it is possible that the proposed anti-inflammatory properties of DHA in the gut are reflected in our current observation of a reduction in the incidence of more serious diarrhea during the first year of life.

While the current results add to the understanding of the potential health benefits of LCPUFAs in the infant diet, our study has some important limitations. First, use of the DHA/ARA formula could have been more prevalent in families who were of a higher socioeconomic status. However, differently from our previous study [15], factors such as parents’ educational level, parents’ socioeconomic status, and number of people living in the home were recorded and included as potential covariates in the statistical analysis and proved not to be different between the two groups (Table 1). Furthermore, all French infants have access to free medical care, and the spare economical resources may decrease a potential role of the socioeconomic status in the ability of the families to purchase infant formula.

A second inherent limitation is that the current study was open-label and non-randomized. As such, it is possible that the attending pediatrician may have made recommendations to the parents regarding the use of infant formula prior to study entry. Thus, the pediatrician could have inadvertently introduced a recruitment bias to the current study. However, as it has been previously suggested [29], prospective, observational studies, despite inherent limitations (absence of randomization, unintentional bias, etc.), can provide an important picture of the “real-world” utility of a study product (in this case, infant formula with added DHA/ARA). In spite of the observational nature of this study, the results are consistent with and add to the existing data of randomized, double-blind studies demonstrating an impact of LCPUFAs, including when added to routine infant formulas, on immune health outcomes.

## Conclusions

Adequate intake of DHA and ARA is currently deemed important because of potential beneficial effects on visual acuity and brain development in infants [30], as well as immune health [5]. Expert recommendations exist for the amount of LCPUFA intake for pregnant and lactating women, infants, and children [30-35], and the recommendations for LCPUFAs in infant formula specify that both DHA and ARA should be added [30,31]. Importantly, the levels of DHA and ARA in the infant formula consumed in this study were similar to worldwide means of LCPUFAs in breast milk [32].

The results of this study add to the increasing evidence that DHA and ARA added to infant formula can contribute to improved respiratory health during infancy and childhood. Additionally, dietary intake of DHA and ARA throughout the first year of life may have a positive effect on moderate to severe diarrhea in infants.

## Abbreviations

LCPUFAs: Long chain polyunsaturated fatty acids; DHA; 22:6n-3: Docosahexaenoic acid; ARA; 20:4n-6: Arachidonic acid; ANOVA: Analysis of variance; OR: Odds ratio; HR: Hazard ratio.

## Competing interests

Pr. Lapillonne has no conflicts of interest, personally or financially, in the production or sales of infant formula or nutritional supplements. Pr. Lapillonne has received past honoraria for lecturing from Mead Johnson Nutrition (MJN). Dr. Pastor, Dr. Scalabrin, Dr. Strong, Cheryl Harris, and Weihong Zhuang work in the Department of Medical Affairs at MJN.

## Authors’ contributions

AL conceived and designed the study and interpreted and assessed the data. NP participated in study design and interpreted the data. DS assessed and interpreted the data and helped draft the manuscript. WZ conducted the statistical analysis and interpreted the data. All authors read and approved the final manuscript.

## Pre-publication history

The pre-publication history for this paper can be accessed here:

http://www.biomedcentral.com/1471-2431/14/168/prepub

## References

[B1] Gottrand F Long-chain polyunsaturated fatty acids influence the immune system of infants J Nutr 2008 138 1807S 1812S 18716191 10.1093/jn/138.9.1807S

[B2] Calder PC Omega-3 fatty acids and inflammatory processes Nutrients 2010 2 355 374 22254027 10.3390/nu2030355 PMC3257651

[B3] Field CJ Thomson CA Van Aerde JE Parrott A Euler A Lien E Clandinin MT Lower proportion of CD45R0+ cells and deficient interleukin-10 production by formula-fed infants, compared with human-fed, is corrected with supplementation of long-chain polyunsaturated fatty acids J Pediatr Gastroenterol Nutr 2000 31 291 299 10997375 10.1097/00005176-200009000-00017

[B4] Field CJ Van Aerde JE Robinson LE Clandinin MT Effect of providing a formula supplemented with long-chain polyunsaturated fatty acids on immunity in full-term neonates Br J Nutr 2008 99 91 99 17640422 10.1017/S0007114507791845

[B5] Hageman JH Hooyenga P Diersen-Schade DA Scalabrin DM Wichers HJ Birch EE The impact of dietary long-chain polyunsaturated fatty acids on respiratory illness in infants and children Curr Allergy Asthma Rep 2012 12 564 573 23001718 10.1007/s11882-012-0304-1 PMC3492691

[B6] Hoppu U Rinne M Lampi AM Isolauri E Breast milk fatty acid composition is associated with development of atopic dermatitis in the infant J Pediatr Gastroenterol Nutr 2005 41 335 338 16131990 10.1097/01.mpg.0000168992.44428.fa

[B7] Thijs C Muller A Rist L Kummeling I Snijders BE Huber M van Ree R Simoes-Wust AP Dagnelie PC van den Brandt PA Fatty acids in breast milk and development of atopic eczema and allergic sensitisation in infancy Allergy 2011 66 58 67 20659079 10.1111/j.1398-9995.2010.02445.x

[B8] Manley BJ Makrides M Collins CT McPhee AJ Gibson RA Ryan P Sullivan TR Davis PG High-dose docosahexaenoic acid supplementation of preterm infants: respiratory and allergy outcomes Pediatrics 2011 128 e71 77 21708809 10.1542/peds.2010-2405

[B9] Alm B Aberg N Erdes L Mollborg P Pettersson R Norvenius SG Goksor E Wennergren G Early introduction of fish decreases the risk of eczema in infants Arch Dis Child 2009 94 11 15 18818269 10.1136/adc.2008.140418 PMC2597687

[B10] Hesselmar B Saalman R Rudin A Adlerberth I Wold A Early fish introduction is associated with less eczema, but not sensitization, in infants Acta Paediatr 2010 99 1861 1867 20670305 10.1111/j.1651-2227.2010.01939.x

[B11] Oien T Storro O Johnsen R Do early intake of fish and fish oil protect against eczema and doctor-diagnosed asthma at 2 years of age? A cohort study J Epidemiol Community Health 2010 64 124 129 19666630 10.1136/jech.2008.084921

[B12] Virtanen SM Kaila M Pekkanen J Kenward MG Uusitalo U Pietinen P Kronberg-Kippila C Hakulinen T Simell O Ilonen J Veijola R Knip M Early introduction of oats associated with decreased risk of persistent asthma and early introduction of fish with decreased risk of allergic rhinitis Br J Nutr 2010 103 266 273 19674492 10.1017/S0007114509991541

[B13] Nafstad P Nystad W Magnus P Jaakkola JJ Asthma and allergic rhinitis at 4 years of age in relation to fish consumption in infancy J Asthma 2003 40 343 348 12870829 10.1081/jas-120018633

[B14] Goksor E Alm B Thengilsdottir H Pettersson R Aberg N Wennergren G Preschool wheeze - impact of early fish introduction and neonatal antibiotics Acta Paediatr 2011 100 1561 1566 21767307 10.1111/j.1651-2227.2011.02411.x

[B15] Pastor N Soler B Mitmesser SH Ferguson P Lifschitz C Infants fed docosahexaenoic acid- and arachidonic acid-supplemented formula have decreased incidence of bronchiolitis/bronchitis the first year of life Clin Pediatr 2006 45 850 855 10.1177/1073858406289801 17041174

[B16] WHO WHO Child Growth Standards: length/Height-for-age, weight-for-age, weight-for-length, weight-for-height and body mass index-for-age. Methods and development WHO Child Growth Standards: Length/Height-for-age, weight-for-age, weight-for-length, weight-for-height and body mass index-for-age 2006 WHO Methods and development (accessed online June 2014: http://www.who.int/childgrowth/standards/technical_report/en/)

[B17] Clark JP How to Peer Review A Qualitative Manuscript 2003 2 London: BMJ Books

[B18] Birch EE Khoury JC Berseth CL Castaneda YS Couch JM Bean J Tamer R Harris CL Mitmesser SH Scalabrin DM The impact of early nutrition on incidence of allergic manifestations and common respiratory illnesses in children J Pediatr 2010 156 902 906 906 e901 20227721 10.1016/j.jpeds.2010.01.002

[B19] Minns LM Kerling EH Neely MR Sullivan DK Wampler JL Harris CL Berseth CL Carlson SE Toddler formula supplemented with docosahexaenoic acid (DHA) improves DHA status and respiratory health in a randomized, double-blind, controlled trial of US children less than 3 years of age Prostaglandins Leukot Essent Fatty Acids 2010 82 287 293 20207123 10.1016/j.plefa.2010.02.009

[B20] Thienprasert A Samuhaseneetoo S Popplestone K West AL Miles EA Calder PC Fish oil n-3 polyunsaturated fatty acids selectively affect plasma cytokines and decrease illness in Thai schoolchildren: a randomized, double-blind, placebo-controlled intervention trial J Pediatr 2009 154 391 395 18930251 10.1016/j.jpeds.2008.09.014

[B21] Haworth O Levy BD Endogenous lipid mediators in the resolution of airway inflammation Eur Respir J 2007 30 980 992 17978156 10.1183/09031936.00005807 PMC3005702

[B22] Serhan CN Chiang N Van Dyke TE Resolving inflammation: dual anti-inflammatory and pro-resolution lipid mediators Nat Rev Immunol 2008 8 349 361 18437155 10.1038/nri2294 PMC2744593

[B23] Serhan CN Yang R Martinod K Kasuga K Pillai PS Porter TF Oh SF Spite M Maresins: novel macrophage mediators with potent antiinflammatory and proresolving actions J Exp Med 2009 206 15 23 19103881 10.1084/jem.20081880 PMC2626672

[B24] D'Vaz N Meldrum SJ Dunstan JA Lee-Pullen TF Metcalfe J Holt BJ Serralha M Tulic MK Mori TA Prescott SL Fish oil supplementation in early infancy modulates developing infant immune responses Clin Exp Allergy 2012 42 1206 1216 22805468 10.1111/j.1365-2222.2012.04031.x

[B25] Furuhjelm C Warstedt K Larsson J Fredriksson M Bottcher MF Falth-Magnusson K Duchen K Fish oil supplementation in pregnancy and lactation may decrease the risk of infant allergy Acta Paediatr 2009 98 1461 1467 19489765 10.1111/j.1651-2227.2009.01355.x

[B26] Imhoff-Kunsch B Stein AD Martorell R Parra-Cabrera S Romieu I Ramakrishnan U Prenatal docosahexaenoic acid supplementation and infant morbidity: randomized controlled trial Pediatrics 2011 128 e505 512 21807696 10.1542/peds.2010-1386 PMC3164093

[B27] Thomas T Eilander A Muthayya S McKay S Thankachan P Theis W Gandhe A Osendarp SJ Kurpad AV The effect of a 1-year multiple micronutrient or n-3 fatty acid fortified food intervention on morbidity in Indian school children Eur J Clin Nutr 2012 66 452 458 22009072 10.1038/ejcn.2011.178

[B28] Teitelbaum JE Allan Walker W Review: the role of omega 3 fatty acids in intestinal inflammation J Nutr Biochem 2001 12 21 32 11179858 10.1016/s0955-2863(00)00141-8

[B29] Kiri VA A pathway to improved prospective observational post-authorization safety studies Drug Saf 2012 35 711 724 22861669 10.1007/BF03261968

[B30] Hoffman DR Boettcher JA Diersen-Schade DA Toward optimizing vision and cognition in term infants by dietary docosahexaenoic and arachidonic acid supplementation: a review of randomized controlled trials Prostaglandins Leukot Essent Fatty Acids 2009 81 151 158 19505812 10.1016/j.plefa.2009.05.003

[B31] AFSSA Opinion of the French Food Safety Agency on the update of French population reference intakes (ANCs) for fatty acids 2010 (accessed online June 2014: http://www.anses.fr/en/content/opinion-french-food-safety-agency-update-french-population-reference-intakes-ancs-fatty)

[B32] Brenna JT Lapillonne A Background paper on fat and fatty acid requirements during pregnancy and lactation Ann Nutr Metab 2009 55 97 122 19752538 10.1159/000228998

[B33] FAO Fats and fatty acids in human nutrition: report of an expert consultation Book fats and fatty acids in human nutrition: report of an expert consultation 2010 1 189 (accessed online June 14: http://www.who.int/nutrition/publications/nutrientrequirements/fatsandfattyacids_humannutrition/en/) 21812367

[B34] Uauy R Dangour AD Fat and fatty acid requirements and recommendations for infants of 0–2 years and children of 2–18 years Ann Nutr Metab 2009 55 76 96 19752537 10.1159/000228997

[B35] EFSA NDA Panel Scientific substantiation of a health claim related to docosahexaenoic acid (DHA) and arachidonic acid (ARA) and visual development pursuant to Article14 of Regulation (EC) No 1924/20061 EFSA J 2009 941 1 14

